# Baseline associations between thoracic expansion, respiratory function, and pain in nonspecific low back pain: A cross-sectional analysis from the DIAFRAGMA trial

**DOI:** 10.1371/journal.pone.0346124

**Published:** 2026-04-03

**Authors:** Francisco José Vera-Serrano, Maria Jesus Vinolo-Gil, María Rebollo-Ramos, María-José Estebanez-Pérez, José-Manuel Pastora-Bernal, Juan Antonio Díaz-Mancha

**Affiliations:** 1 Physiotherapy Department, Faculty of Physiotherapy and Podiatry, University of Seville, Seville, Spain; 2 Department of Nursing and Physiotherapy, Faculty of Nursing and Physiotherapy, University of Cádiz, Cádiz, Spain; 3 Research Unit, Biomedical Research and Innovation Institute of Cádiz (INiBICA), Puerta del Mar University Hospital, University of Cádiz, Cádiz, Spain; 4 Rehabilitation Clinical Management Unit, Interlevels-Intercenters Hospital Puerta del Mar, Hospital Puerto Real, Cádiz, Spain; 5 ExPhy Research Group, Department of Nursing and Physiotherapy, Instituto de Investigación e Innovación Biomédica de Cádiz (INiBICA), Universidad de Cádiz, Cádiz, Spain; 6 Department of Physiotherapy, Faculty of Health Sciences, University of Málaga, Málaga, Spain; Marwadi University, INDIA

## Abstract

**Objective:**

To examine the associations of respiratory variables (chest and abdominal expansion, spirometry) with pain, lumbar mobility, and quality of life in nonspecific low back pain (NSLBP) using baseline data from the DIAFRAGMA trial.

**Design:**

Cross-sectional study using baseline data from a randomized controlled trial.

**Participants:**

Fifty subjects (22 females [44%], 28 males [56%]; mean age 45.96 years; BMI 26.97 kg/m²) with NSLBP recruited between January and July 2024. Main measures: Axillary, sternal, and abdominal inspiratory expansion; spirometric parameters (FVC, FEV1); pain via pressure pain thresholds (PPT), Schober test, FFT; and quality of life via SF-12.

**Methods:**

Pearson correlations assessed relationships between respiratory and clinical variables. Multiple linear regression adjusted for age, sex, and BMI. Significance was set at p < 0.05 with Bonferroni correction.

**Results:**

Abdominal expansion correlated with FVC (r = 0.45; p < 0.001), FEV1 (r = 0.39; p = 0.003), PEF (r = 0.28; p = 0.03), axillary (r = 0.47; p < 0.001) and sternal expansion (r = 0.58; p < 0.001), PPT at L4 (r = 0.26; p = 0.04) and L5 (r = 0.29; p = 0.02), and SF-12 (r = 0.25; p = 0.04). BMI correlated negatively (r = –0.31; p = 0.02). In regression, abdominal expansion independently predicted pain after adjustment.

**Conclusions:**

Reduced thoracoabdominal expansion and spirometric performance are associated with greater pain and poorer quality of life in NSLBP, supporting the diaphragm’s role in low back pain.

## Introduction

The increasing prevalence of low back pain in the current population is primarily associated with repetitive trauma and overuse, which are common causes of mechanical low back pain, particularly in the workplace [1]. It is noteworthy that most individuals with limiting low back pain tend to experience recurrent episodes, which favors the progression to chronicity [2] Chronic low back pain affects up to 23% of the global population, and it is estimated that between 24% and 80% of these patients experience recurrences, resulting in significant functional limitations and dependence, especially in older age [3,4]. Nonspecific low back pain (NSLBP) remains one of the leading causes of disability globally. Emerging evidence indicates that diaphragmatic function, chest expansion, and respiratory parameters may influence spinal stability, intra-abdominal pressure, and consequently pain in NSLBP patients. The systematic review by Fabero-Garrido et al. [5] demonstrated that respiratory muscle training significantly improves pain and lung function in individuals with LBP.

As a result, this condition has become one of the leading causes of healthcare expenditure, with significant increases in procedures such as epidural corticosteroid injections (629%) and opioid prescriptions (423%). Furthermore, increasing use of magnetic resonance imaging and spinal fusion surgeries have been observed. However, this increase in costs does not necessarily translate into improved clinical outcomes, as in many cases, treatments are insufficient or inadequate, and disability rates associated with low back pain remain high [6].

Spinal stability depends on various structural elements, such as the intervertebral discs, ligaments (which provide intrinsic stability), and adjacent musculature (responsible for extrinsic stability) [7]. Denis model [8], widely accepted in biomechanics and traumatology, proposes a functional classification of the spine into three columns, each with a specific role in its stability and dynamics. Among the extrinsic elements with potential influence on lumbar spine stabilization is the diaphragm muscle [9]. The lumbar portion of the diaphragm originates from the medial, intermediate, and lateral diaphragmatic pillars and establishes anatomical relationships with the retropericardial and perinephric structures, as well as with their associated fat [10]. The right medial pillar, which is more robust and extensive, becomes a flat tendon that inserts into the anterior surface of the L2-L3 vertebrae and, occasionally, L4 [11]. Adjacent to this is the accessory or intermediate pillar, whose tendon extends between the L1 and L2 vertebrae. The left medial pillar ends as a flat tendon that inserts between L2 and L3. The lateral pillars extend into two main ligaments: the medial arcuate ligament (which lies superior to the psoas muscle and connects the L1 vertebra to its transverse process) and the lateral arcuate ligament (located over the quadratus lumborum muscle, connecting the transverse process of L1 to the end of the twelfth rib) [12], and the lateral arcuate ligament (located superior to the quadratus lumborum muscle), which connects the transverse process of L1 to the apex of the twelfth rib [13]. Both ligaments act as structural bridges between the posterior thoracolumbar fascia and the anterior transversalis fascia [14,15], thus contributing to the diaphragm's ability to function as a stabilizer of the lumbar spine.

According to van Dam van Isselt et al. [16], the lumbar region is the most affected in patients presenting with pain accompanied by respiratory dysfunction. In young, middle-aged subjects with nonspecific low back pain, morphological and structural alterations in diaphragm muscle thickness have been identified, both during inspiration and expiration [17]. Despite the diaphragm's dual role in respiration and trunk stability, knowledge about its bilateral function in subjects with low back pain is still limited. In this regard, Ziaeifar et al. [18], demonstrated, using ultrasound, changes in the thickness of the right hemidiaphragm, which could significantly predict the presence of low back pain.

Likewise, Martí-Salvador et al. [19], demonstrated that an osteopathic treatment protocol that includes specific diaphragm techniques generates significant and clinically relevant improvements in pain and disability in patients with low back pain. In the field of sports, Sannasi R et al. [20], emphasize the importance of including exercises aimed at facilitating physiological breathing patterns and progressively restoring postural control of the diaphragm within lumbar stabilization programs for elite athletes. In addition, Zhou et al [21], showed that weightlifters with chronic low back pain have reduced diaphragmatic contractility, which is associated with lower lifting performance.

Prior pilot studies, such as Naranjo-Cinto et al [22], have shown morphological and functional differences in diaphragm between those with and without NLBP. However, baseline cross-sectional associations between chest/abdominal expansion, spirometric measures, and clinical parameters like pain, mobility, and quality of life have been less explored with robust multivariable adjustments. The current analysis of baseline DIAFRAGMA data aims to fill this gap.

## Materials and methods

### Study design

Cross-sectional analysis using baseline data from the DIAFRAGMA randomized controlled trial. The research adhered to the principles outlined in the Declaration of Helsinki for ethical practices involving human participants. Approval was granted by the Research Ethics Committee of the University of Seville, Spain (ref. 1032-N-23). All participants provided written informed consent prior to data collection.

### Participants

Fifty adults with nonspecific low back pain (22 females, 28 males), aged 18–74 years, with a mean Body mass index (BMI) 26.97 ± 5.15 kg/m^2^were prospectively recruited from a private physiotherapy clinic (“Fisioclinic”) between 01/01/2024 and 30/07/2024, corresponding to the period of formal participant enrollment.

An external assistant, independent from the research team, informed potential participants orally about the study. Participation was voluntary, and participants could refuse at any time. Those who agreed to participate received a written information sheet and provided written informed consent, which was collected and securely stored. Subjects who signed the consent were subsequently included in the randomization process.

Participants were eligible if they were over 18 years old who fulfilled the eligibility criteria. Inclusion criteria: diagnosis of subacute or chronic NSLBP by a specialist, attendance at the “Fisioclinic” Physiotherapy Clinic, age over 18 years, and provision of signed informed consent. Exclusion criteria: history of surgical intervention on the upper or lower limbs, head, spine, thorax, or abdomen; imaging-confirmed anatomical abnormalities; non-mechanical pathologies (inflammatory, infectious, tumorous, neurological, traumatic, or bone diseases of the lumbar spine); analgesic or anti-inflammatory treatment within two weeks prior to the study; pregnancy or breastfeeding; chemotherapy or radiotherapy; systemic rheumatic diseases (e.g., arthritis, osteoarthritis, gout, psoriasis); implanted electronic devices; substance abuse; use of central nervous system-affecting medications (e.g., antidepressants, anxiolytics, anticonvulsants); prior experience with diaphragmatic manual treatment; high physical work activity; age outside the study range; or refusal to participate and sign informed consent.

Diaphragmatic function and respiratory parameters were assessed at baseline as outcome variables to explore their potential association with pain and lumbar mobility. No screening for diaphragmatic dysfunction was performed prior to inclusion; participants were enrolled based solely on the presence of NSLBP, regardless of respiratory status. This approach allowed examination of the relationship between diaphragmatic function and low back pain in an unselected NSLBP population.

The data analyzed in this study correspond to the baseline assessments of the DIAFRAGMA randomized controlled trial, conducted between 01/01/2024 and 30/07/2024. In the main trial, participants were later randomly assigned to either a control group (conventional physiotherapy) or an experimental group (conventional physiotherapy plus manual diaphragmatic treatment).

### Outcome measures

#### Measurement of thoracic expansion with cirtometry.

It can be used as a method of evaluating the expansion of diaphragmatic breathing to quantify possible alterations in thoracic capacity and abdominal and chest wall compliance achieved by all expiratory and inspiratory muscles. These measurements showed good intra- and interrater reliability and reproducibility (intraclass correlation coefficient (ICC), Intra-rater 0.92 and Inter-rater 0.85 with correlation in each other (r = 0.747; P < .01)) [23]. By recording the expansion of the rib cage with a tape measure over the second intercostal space (axillary level), the xiphoid process (sternal level), and the midpoint between the xiphoid process and the umbilicus (abdominal level), proficiency in diaphragmatic breathing can be demonstrated. With the patient standing and with his hands on his head, the patient was asked to “inhale as much as possible” and “exhale as much as possible.” Maximum inspiration and expiration to obtain the thoracic-abdominal amplitude coefficient, which is characterized by the difference between these values in the axilla (CA-Ax), xiphoid (CA-Xif) and basal (CA-Ba) [24]. To ensure consistent thoracic circumferential measurements, standardized anatomical landmarks were used: the fourth intercostal space anteriorly and the subscapular region posteriorly [23,24]. A sweat-resistant dermal marker maintained visible reference points across sessions. Tape alignment was checked before each measurement, and a non-elastic tape ensured accuracy without stretching. The same trained evaluator conducted all sessions, applying consistent tension without compressing tissues, minimizing variability. Repeated measurements were averaged to reduce error. Respiratory phases (maximum inspiration, expiration, and resting) were measured separately to ensure comparability and to differentiate the roles of inspiratory and expiratory muscles in thoracic expansion at each reference level.

#### Measurement of respiratory parameters FEV1 and FVC through spirometry with the CONTEC SPIROMETER SP10W device.

The inter-rater reliability of the pulmonary function test is r = .99. [25]. For validation and accuracy of the tests, quality control of the equipment with periodic calibration checks was performed before testing, according to the recommendations of the American Thoracic Society [26,27]. First, the subject is placed in a sitting position, the patient holds the spirometer horizontally in his right hand with the screen pointing upwards. He places the spirometer close to his mouth and presses the operating button. A short beep will be heard. At the second beep, the subject takes in as much air as he can. He then places the mouthpiece of the spirometer in his mouth and blows as hard as he can for at least 1.5 seconds. The measurement will be repeated at least twice. One-minute intervals will be applied between these measurements to avoid short-term respiratory muscle fatigue [26,28].

#### Measurement of lumbar mobility through the Schober Test.

The validity against radiographs was strong (r = 0.90) [29]. The interclass (r = 0.90) and intraclass (r = 0.96) reliability was found to be excellent. This test has an ICC of 0.60–0.97 [23,30]. With the patient standing and arms at the sides, the therapist, positioned laterally, marked the skin at the level of S1 and 10 cm above, using the PSIS as a reference to identify S1. This method, validated in clinical practice, offers good inter-rater reliability. The patient was instructed to flex and extend the trunk while measuring the distance between the two marks. Normal values are 15 cm in flexion and 8–9 cm in extension; reduced ranges suggest lumbar, coxofemoral, or hamstring restrictions. A trained evaluator performed all measurements, repeating them for consistency and averaging values. Skin markings were reinforced with a dermatographic pencil, ensuring anatomical accuracy across sessions.

#### Measurement of lumbar mobility through FFT.

This test showed strong reliability, with an ICC of 0.999 (95% CI, 0.998–1.00) [31]. In a previous study [2], it was found that a restriction in hamstring range of motion could be associated with an increased risk of developing LBP. With the patient standing with feet together and knees fully extended, the therapist squatted to one side of the patient so that the resulting measurement could be made with the help of a tape measure. The therapist asked the patient to pre-flex the spine so that his arms, hands and fingers were fully extended and relaxed forward. To carry out the anterior flexion of the spine, the patient actively performed a flexion of the hips bilaterally, a flexion of the lumbar spine following the one where the hip began, especially the L5-S1 and, finally, placement of the pelvis in retreat so that the flexion of the spine reaches its maximum amplitude. All measurements were conducted following a strict protocol, ensuring that participants maintained the same stance, posture, and movement sequence at every time point. The test was performed at a consistent time of day to minimize diurnal variability in flexibility. The patients were instructed to slowly bend forward without actively stretching the hamstrings beyond their natural range. No warm-up or stretching exercises were allowed before testing to avoid temporary gains in flexibility. Hip movement was kept consistent by instructing participants to flex forward only from the lumbar spine, minimizing pelvic rotation. Also, measures were repeated twice, and the mean value was used. Both Schober’s measurement and FFT were recorded simultaneously to evaluate possible discrepancies between lumbar mobility and the distance of the fingers from the floor.

#### Measurement of the pressure pain threshold (PPT) of the five lumbar vertebrae and lumbar paravertebral muscles using the Trigger Plus, Palpatronic, Hagen, Germany.

This tool has demonstrated good intra-examiner reliability [32,33]. It is a reliable, sensitive and reproducible instrument to assess PPT at the center of the paravertebral spinal musculature bilaterally and perpendicular to the spinous process of L3 [34]. PPT measurements were applied with pressure at a rate of 10 N/cm2/s until the participant first reported a painful sensation [32] both in the paravertebral musculature at the level of right and left L3 and in all spinous processes from L1-L5. This process will be performed twice in the same place and within an interval of 30–60 s, using the average of these two measurements [34,35].

### Data analysis

The data were analyzed with the statistical package Statistical Package for the Social Sciences (SPSS) Statistic® software for Windows version 26. Descriptive analysis: absolute frequency, mean values, standard deviation, minimum and maximum were calculated. Inferential analysis: 95% confidence levels were considered, so the experimental p-value had a significant level of 5%. Normality tests: Normal distribution of data was assessed with the Shapiro-Wilk test.

A Pearson correlation analysis examines the relationships between the studied variables, aiming to determine potential associations between thoracic expansion, lumbar mobility, pain and respiratory parameters in patients with NSLBP.

Multiple linear regression models with independent variables including expansions and spirometric parameters; dependent variables: pain, mobility, quality of life; adjusted for age, sex, BMI. Bonferroni correction for multiple tests. To identify independent predictors of abdominal inspiratory expansion, multiple linear regression analyses were performed. In the main model abdominal inspiratory expansion was entered as the dependent variable and age, sex, BMI, FVC, FEV1, PEF, Schober flexion-extension, FFT, mean PPT, inspiratory expansion and SF-12 as independent variables. Regression assumptions were checked: normality of residuals (histogram and P-P plot), homoscedasticity (residuals vs predicted plot) and independence (Durbin-Watson). Multicollinearity was assessed via VIF and tolerance, with VIF > 5 indicating concern. We report unstandardized (B) and standardized (β) coefficients, standard errors (SE), 95% CI and p-values. Two-tailed tests were used and significance set at p < 0.05.

## Results

### Sample size

A total of 50 participants with nonspecific low back pain (22 women, 44%; 28 men, 56%), aged between 18 and 74 years (mean = 45.96 ± 16.07 years) and with a body mass index (BMI) of 26.97 ± 5.15 kg/m², were included in the analysis.

This analysis corresponds to the baseline assessments of the DIAFRAGMA randomized controlled trial, conducted between January and July 2024, prior to any intervention. The sample consisted of 50 participants with no data loss during baseline evaluations. Demographic and clinical characteristics are presented in Table 1.

**Table 1:** Demographic characteristics of the sample.

Variable	Mean ± SD / N (%)	Range / 95% CI
Age, years	45.96 ± 16.07	18 - 74
Sex (female / male)	22 (44%) / 28 (56%)	
Height, cm	173.40 ± 9.04	154 −198
Weight, kg	78.72 ± 17.76	52 −132
Body Mass Index, kg/cm^2^	26.97 ± 5.15	17.78–46.22
Axillar inspiratory expansion, cm	2.18 ± 0.82	0.50–4.75
Abdominal inspiratory expansion, cm	2.82 ± 1.37	0.50–6.75
Sternal inspiratory expansion, cm	3.54 ± 1.47	0.50–6.50
Schober Flexion Test, cm	14.59 ± 1.18	11.25–17.25
Schober Extension Test, cm	10.02 ± 0.48	9.00–11.25
FFT Test, cm	17.64 ± 10	0–44.5
PPT L1, Kg/cm^2^	6.83 ± 1.93	3.50–10.00
PPT L2, Kg/cm^2^	6.88 ± 1.91	3.00–10.00
PPT L3, Kg/cm^2^	6.69 ± 1.94	3.50–10.00
PPT L4, Kg/cm^2^	6.44 ± 1.99	3.00–10.00
PPT L5, Kg/cm^2^	6.23 ± 1.93	3.00–10.00
PPT Right PV, Kg/cm^2^	7.18 ± 1.94	2.25–10.00
PPT Left PV, Kg/cm^2^	7.25 ± 1.89	2.75–10.00
FVC, l	3.90 ± 1.04	2.08–5.89
FEV1, l	3.02 ± 0.90	1.31–4.73
PEF, l	5.57 ± 2.84	1.22–12.21
SF-12, p	774.70 ± 227.90	320 - 1150

Values expressed as mean ± standard deviation (SD) or number (percentage). All data corresponds to baseline (pre-intervention) assessments. Abbreviations: N, number of patients.

### Main findings

Pearson correlation analysis was conducted to examine the relationship between inspiratory thoracic expansion, respiratory function with forced vital capacity (FVC), forced expiratory volume in one second (FEV1), and peak expiratory flow (PEF). Lumbar mobility with Schober test in flexion, in extension and FFT test. Finally, PPT in all different points and SF-12.

Throughout the study, abdominal inspiratory expansion correlated significantly with FVC (r = 0.45; p < 0.001), FEV1 (r = 0.39; p = 0.003), PEF (r = 0.28; p = 0.03). Also correlated with axillar and sternal inspiratory expansion (r = 0.47; p < 0.001 and r = 0.58; p < 0.001 respectively). PPT was significant in L4 (r = 0.257; p = 0.04) and in L5 (r = 0.294; p = 0.02). Finally, also with SF-12 questionnaire (r = 0.25; p = 0.04). BMI was significant (r = −0.31; p = 0.02) (Tables 2–6). After Bonferroni correction, associations with FVC and FEV1 remained significant.

**Table 2:** Correlation matrix between abdominal inspiratory expansion and clinical and respiratory variables at baseline (N = 50). Pearson’s correlation coefficients (r) are shown. Statistically significant correlations (p < 0.05) are highlighted in bold.

Pearson			Abdominal inspiratory expansion	Age	BMI	Schober Flexion Test
Abdominal inspiratory expansion	Pearson’s correlation coefficients (r)	1.000	−.16	−.31	.01
	Sig.(two-sided)	.	.14	**.02**	.47
Age	Pearson’s correlation coefficients (r)	−.16	1.000	.13	−.08
	Sig.(two-sided)	.14	.	.19	.29
BMI	Pearson’s correlation coefficients (r)	−.31	.13	1.000	.11
	Sig.(two-sided)	.02	.19	.	.22
Schober Flexion Test	Pearson’s correlation coefficients (r)	.01	−.08	.11	1.000
	Sig.(two-sided)	.47	.29	.22	.

**Table 3:** Correlation matrix between abdominal inspiratory expansion and clinical and respiratory variables at baseline (N = 50). Pearson’s correlation coefficients (r) are shown. Statistically significant correlations (p < 0.05) are highlighted in bold.

Pearson			Abdominal inspiratory expansion	Schober extensión Test	PPT L1	PPT L2	PPT L3
Abdominal inspiratory expansion	Pearson’s correlation coefficients (r)	1.000	.11	.01	.02	.15
	Sig.(two-sided)	.	.22	.47	.44	.15
Schober extension Test	Pearson’s correlation coefficients (r)	.11	1.000	.00	−.14	−.12
	Sig.(two-sided)	.22	.	.50	.18	.20
PPT L1	Pearson’s correlation coefficients (r)	.01	.00	1.000	.85	.61
	Sig.(two-sided)	.47	.50	.	**<.001**	**<.001**
PPT L2	Pearson’s correlation coefficients (r)	.02	−.14	.85	1.000	.80
	Sig.(two-sided)	.44	.18	**<.001**	.	**<.001**
PPT L3	Pearson’s correlation coefficients (r)	.15	−.12	.61	.80	1.000
	Sig.(two-sided)	.15	.50	**<.001**	**<.001**	.

**Table 4:** Correlation matrix between abdominal inspiratory expansion and clinical and respiratory variables at baseline (N = 50). Pearson’s correlation coefficients (r) are shown. Statistically significant correlations (p < 0.05) are highlighted in bold.

Pearson			Abdominal inspiratory expansion	PPT L4	PPT L5	PPT Right PV	PPT Left PV
Abdominal inspiratory expansion	Pearson Correlation	1.000	.26	.29	.12	.23
	Sig.(two-sided)	.	**.04**	**.02**	.20	.06
PPT L4	Pearson’s correlation coefficients (r)	.26	1.000	.76	.64	.56
	Sig.(two-sided)	**.04**	.	**<.001**	**<.001**	**<.001**
PPT L5	Pearson’s correlation coefficients (r)	.29	.76	1.000	.73	.51
	Sig.(two-sided)	**.02**	**<.001**	.	**<.001**	**<.001**
PPT Right PV	Pearson’s correlation coefficients (r)	.12	.64	.73	1.000	.79
	Sig.(two-sided)	.20	**<.001**	**<.001**	.	**<.001**
PPT Left PV	Pearson’s correlation coefficients (r)	.23	.56	.51	.79	1.000
	Sig.(two-sided)	.06	**<.001**	**<.001**	**<.001**	.

**Table 5:** Correlation matrix between abdominal inspiratory expansion and clinical and respiratory variables at baseline (N = 50). Pearson’s correlation coefficients (r) are shown. Statistically significant correlations (p < 0.05) are highlighted in bold.

Pearson			Abdominal inspiratory expansion	Axilar inspiratory expansion	Sternal inspiratory expansion	FFT
Abdominal inspiratory expansion	Pearson’s correlation coefficients (r)	1.000	.47	.58	–.06
	Sig.(two-sided)	.	<.001	<.001	.33
Axilar inspiratory expansion	Pearson’s correlation coefficients (r)	.47	1.000	.63	.11
	Sig.(two-sided)	<.001	.	<.001	.47
Sternal inspiratory expansion	Pearson’s correlation coefficients (r)	.58	.47	1.000	−.03
	Sig.(two-sided)	<.001	<.001	.	.41
FFT	Pearson’s correlation coefficients (r)	–.06	.11	−.03	1.000
	Sig.(bilateral)	.33	.47	.41	.

**Table 6:** Correlation matrix between abdominal inspiratory expansion and clinical and respiratory variables at baseline (N = 50). Pearson’s correlation coefficients (r) are shown. Statistically significant correlations (p < 0.05) are highlighted in bold.

Pearson			Abdominal inspiratory expansion	FVC	FEV1	PEF	SF-12
Abdominal inspiratory expansion	Pearson’s correlation coefficients (r)	1.000	.46	.39	.28	.25
	Sig.(two-sided)	.	**<.001**	**.003**	**.003**	**.04**
FVC	Pearson’s correlation coefficients (r)	.45	1.000	.80	.48	.17
	Sig.(two-sided)	**<.001**	.	**<.001**	**<.001**	.12
FEV1	Pearson’s correlation coefficients (r)	.39	.80	1.000	.76	.05
	Sig.(two-sided)	**.003**	**<.001**	.	**<.001**	.36
PEF	Pearson’s correlation coefficients (r)	.28	.48	.76	1.000	.09
	Sig.(two-sided)	**.003**	**<.001**	**<.001**	.	.26
SF-12	Pearson’s correlation coefficients (r)	.25	.17	.05	.09	1.000
	Sig.(two-sided)	**.04**	.12	.36	.26	.

The multiple linear regression model including all clinical and respiratory predictors was statistically significant (F (26, 23) = 2.73; p = 0.009), explaining 47.9% of the adjusted variance in abdominal inspiratory expansion (adjusted R² = 0.479; R = 0.869) (Table 7). The standard error of the estimate was 0.99. The Durbin–Watson statistic was 2.50, indicating independence of residuals.

**Table 7:** Multiple linear regression model predicting abdominal inspiratory expansion. Dependent variable: abdominal inspiratory expansion (cm). Values represent unstandardized coefficients (B), standard errors (SE), standardized coefficients (β), 95% confidence intervals (CI), t values and p values.

Predictor	B	SE	β	t	p	CI (95%)
Age	−0.01	0.02	−0.05	−0.29	0.78	(−0.04-0.03)
BMI	−0.10	0.05	−0.39	−2.17	0.04	(−0.20-(−0.01))
SCHOBER FLEXION	0.14	0.17	0.13	0.86	0.40	(−0.20-0.49)
SCHOBER EXTENSION	−0.19	0.46	−0.01	−0.04	0.968	(−0.96-0.93)
FFT	0.01	0.02	0.06	0.47	0.65	(−0.03-0.04
PPT L1	0.04	0.23	0.06	0.18	0.86	(−0.43–0.51)
PPT L2	−0.35	0.32	−0.49	−1.10	0.28	(−1.01–0.31)
PPT L3	0.51	0.23	0.72	2.22	0.04	(0.03–0.98)
PPT L4	−0.13	0.22	−0.19	−0.60	0.55	(−0.59–0.32)
PPT L5	0.01	0.21	0.01	0.04	0.97	(−0.42–0.44)
PPT PV R	−0.12	0.22	−0.15	−0.47	0.64	(−0.57–0.36)
PPT PV L	0.10	0.18	0.14	0.57	0.58	(−0.27–0.48)
AXILAR INS	0.33	0.36	0.20	0.91	0.37	(−0.42–1.09)
STERNAL INS	−0.04	0.25	−0.05	−0.17	0.87	(−0.56–0.47)
FVC	0.38	0.48	0.29	0.78	0.44	(−0.62–1.37)
FEV1	0.42	0.71	0.28	0.59	0.56	(−1.04–1.88)
PEF	−0.40	0.28	−0.84	--1.41	0.17	(−0.99–0.19)
SF-12	−0.001	0.001	−0.11	−0.70	0.49	(−0.003–0.001)

**Fig 1:**
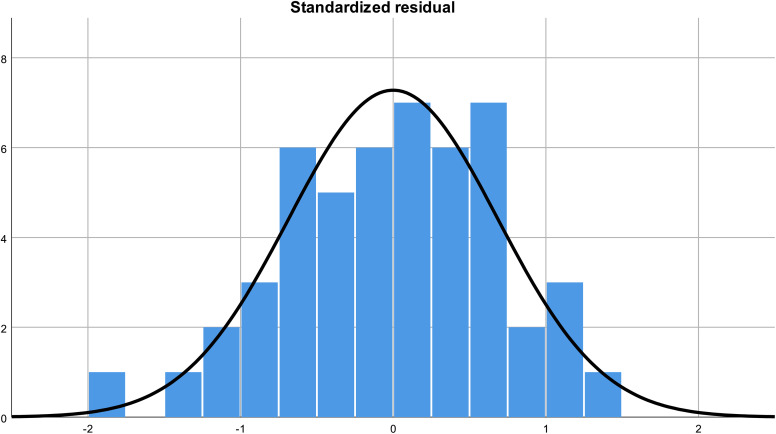
Histogram of standardized residuals from the multiple linear regression model.

**Fig 2:**
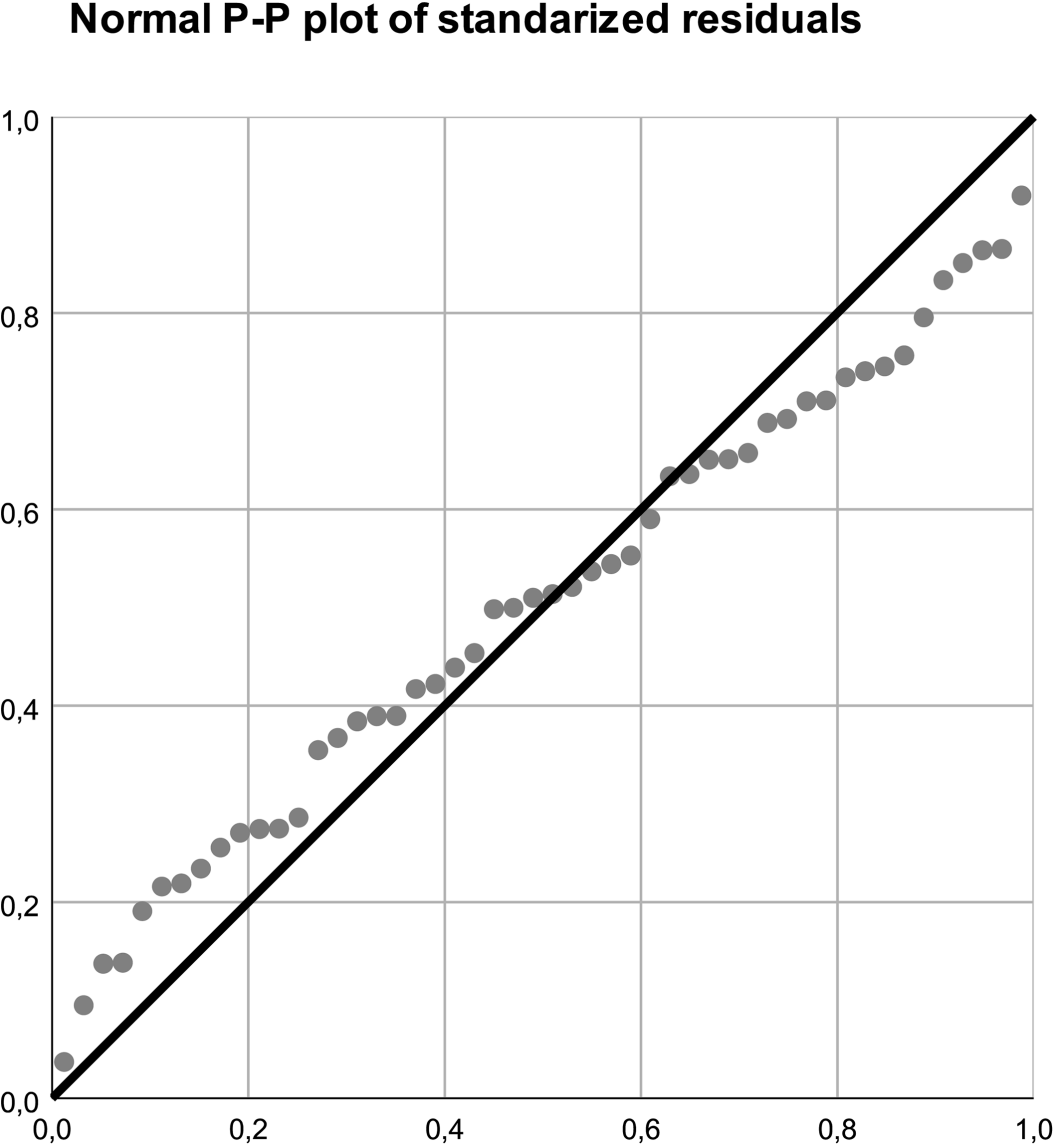
Normal P–P plot of standardized residuals for the multiple regression model.

In the final model, abdominal inspiratory expansion was independently associated with body mass index (BMI) (B = –0.10, β = –0.39, 95% CI [–0.20, –0.01], p = 0.04) and with lumbar pressure pain threshold at L3 (PPT L3) (B = 0.51, β = 0.72, 95% CI [0.03, 0.98], p = 0.04). No major multicollinearity issues were detected (VIF range: 0.5–4.2). Assumptions of linearity, homoscedasticity, and normality of residuals were checked. The histogram of standardized residuals and the normal P–P plot confirmed that residuals were approximately normally distributed and randomly dispersed around zero (Figs 1 and 2).

These findings suggest that a lower BMI and higher lumbar PPT values are associated with greater abdominal inspiratory expansion at baseline, indicating a potential link between respiratory mechanics, body composition, and pain sensitivity in patients with nonspecific low back pain. These findings suggest that the set of clinical and respiratory variables significantly predicted abdominal inspiratory expansion, accounting for approximately half of its baseline variability. These findings indicate that greater abdominal inspiratory expansion is associated with improved respiratory efficiency and lower pain sensitivity in NSLBP patients, highlighting the potential diagnostic value of respiratory assessments.

The histogram shows that residuals are approximately normally distributed, supporting the assumption of normality.

The points closely follow the diagonal line, indicating that residuals are normally distributed and the model assumptions are met.

## Discussion

This study aimed to explore associations among pain feeling, respiratory function, and lumbar mobility in individuals with NSLBP at the baseline (pre-intervention) stage. While the primary intent of the original trial was to evaluate the therapeutic effect of a diaphragm-focused intervention, the present cross-sectional analysis provides insight into how these variables relate before any treatment. Our findings underscore meaningful relationships between respiratory mechanics, pain sensitivity, and spinal mobility, supporting the hypothesis that diaphragm function may play a significant role in the multifactorial pathophysiology of NSLBP.

Although our study was cross-sectional, previous longitudinal analyses from the DIAFRAGMA trial suggested that the therapeutic effects of diaphragm-focused interventions tend to accumulate over time [36]. This pattern implies a gradual or sustained adaptation rather than an immediate shift. While the cross-sectional model cannot infer causality, the baseline associations offer plausible mechanistic insight: individuals with more favorable respiratory mechanics and lower pain sensitivity at baseline may respond better to diaphragm-centred interventions.

Anatomical and functional associations between the diaphragm and the lumbopelvic region have been extensively described in the literature. The works of Valenza et al. [37] and González-Álvarez et al. [23] highlight the diaphragm’s contribution to trunk stabilization, findings that resonate with the outcomes observed in the present study. However, inconsistencies across literature may be attributed to heterogeneity in study protocols, including variability in treatment frequency, sample characteristics, and the intensity or type of intervention delivered. The accuracy and reliability of outcome measures are also pivotal in detecting real functional changes. Marizeiro et al. [24] emphasized the relevance of appropriate measurement tools, a point supported here through the effective use of the Schober Test and FFT, which provided objective quantification of lumbar mobility and strengthened the reliability of our results. Notably, the therapeutic value of diaphragmatic techniques extends beyond musculoskeletal disorders. Ahmad et al. [38] demonstrated their efficacy in pulmonary rehabilitation among post-COVID-19 patients. Complementary findings by Rutka et al. [39] and Bennett S et al. [40] observed improved spirometric indices and increased diaphragmatic excursion in children with cerebral palsy, even when the diaphragm was not directly stretched. These effects may be partly explained by patient positioning during the intervention, which, despite not being specifically designed to stretch diaphragmatic fibers, may have facilitated some degree of elongation. Both studies employed cirtometry, enabling consistent and comparable assessment. Further evidence from Alaparthi et al [41] supports the utility of diaphragmatic breathing and volume incentive spirometry in enhancing FVC and diaphragmatic mobility post-laparoscopic surgery. Similarly, Ho et al [42] highlighted the structural importance of the diaphragm in pediatric respiratory function and underscored the potential of ultrasound imaging as a valuable diagnostic and evaluative modality.

In addition to its role in postural and ventilatory mechanics, the diaphragm has also been implicated in pain modulation [9,43]. For instance, Martí-Salvador M et al. [44] observed clinically relevant reductions in pain in MNSLBP patients using subjective pain scales. While their findings suggest positive outcomes, the absence of objective quantification tools such as algometry limits the interpretation of their results. Molina-Hernández N et al. [45], employed inspiratory muscle training and demonstrated significant increases in bilateral PPT in paraspinal musculature, suggesting a possible neuromuscular modulation of the diaphragm affecting pain thresholds. However, their focus remained solely on the paravertebral muscles, without considering the insertional anatomy of the diaphragmatic crura at lumbar vertebral levels. Contrastingly, Marugan-Rubio et al. [34], found no significant group differences in lumbar muscle pain intensity or PPT, highlighting the need for further studies comparing various diaphragmatic interventions to establish which techniques yield measurable improvements in pain sensitivity. Collectively, these findings underscore the diaphragm’s multifaceted role in both respiratory and postural systems and support its incorporation into evidence-based physiotherapeutic management of MNSLBP. [10,46]. Integrating diaphragm-centered interventions could not only enhance clinical outcomes but also improve the overall efficiency of treatment protocols. Revising current clinical guidelines to reflect these insights may be crucial in optimizing care strategies for patients with chronic low back pain. Thus, our correlational results add to this body of evidence by showing that, even at baseline, respiratory and pain-sensitivity metrics are intertwined in patients with low back pain.

Taken together, these findings consolidate the diaphragm’s relevance as a key structure in the interplay between respiratory function and postural control, justifying its inclusion in physiotherapeutic protocols for low back pain. The presence of moderate to strong associations at baseline suggests that clinicians might consider assessing diaphragmatic mobility and respiratory capacity as part of the first evaluation in low back pain. Patients with lower abdominal expansion, lower spirometric indices, or heightened pain sensitivity may benefit most from early respiratory or diaphragm training adjuncts to conventional musculoskeletal therapy. Over time, integrating diaphragm-focused methods might lead to more sustained and multidimensional improvements (mobility, pain, function).

Several limitations must be acknowledged. First, the cross-sectional design precludes any causal inference: we cannot determine whether respiratory dysfunction leads to higher pain or vice versa. Second, baseline-only analysis excludes the dynamic changes over time; hence, temporal causality or mediation cannot be assessed here. Third, potential confounders such as habitual physical activity, smoking history, posture, and psychological factors were not controlled. Finally, measurement limitations may have reduced sensitivity (e.g., cirtometry and algometry have operator-dependent variability), and future studies might improve reliability by including ultrasound, electromyography, or imaging techniques. Despite these limitations, the consistency of associations across respiratory and pain-related domains supports the robustness of our findings. Regarding generalizability, it should be noted that our findings are derived from a general NSLBP population without pre-selection based on respiratory or diaphragmatic status. While this enhances external validity, it also means that the strength of associations observed may differ in subgroups with confirmed diaphragmatic dysfunction. Future studies should investigate whether patients with more pronounced respiratory impairment show stronger correlations or greater responsiveness to diaphragm-focused interventions.

Future studies should adopt prospective or experimental designs to test whether improving diaphragm function causally reduces pain and improves spinal mobility. Comparative trials of different diaphragm interventions (manual stretching, respiratory muscle training, breath retraining) would clarify which modality is most effective and for which patient subgroups. Incorporating advanced measurement techniques (ultrasound diaphragm excursion, EMG, pressure sensors) could deepen mechanistic understanding. Also, controlling lifestyle factors and extending follow-ups beyond 30 days would permit assessment of durability and rebound effects. Future trials should also compare manual, respiratory, and training techniques to identify which yield superior outcomes.

## Conclusions

The present baseline correlational analysis strengthens the conceptual model in which diaphragm function, respiratory mechanics, and pain sensitivity interact in low back pain. These interrelationships justify the inclusion of diaphragm-targeted assessment and therapy within multimodal physiotherapeutic protocols. While our findings are preliminary and correlational, they support the notion that diaphragm performance is a key component of the respiratory–postural matrix that clinicians should not overlook.

## Clinical messages

- Baseline associations in patients with nonspecific low back pain showed that reduced thoracoabdominal inspiratory expansion and lower spirometric performance were linked to greater pain intensity and poorer quality of life.
- Assessing diaphragm-related respiratory function may help identify patients at risk of persistent low back pain or limited recovery.
- Integrating respiratory assessment and diaphragm-focused techniques into physiotherapy programs could improve outcomes beyond conventional lumbar treatment.
- These findings support a multidimensional approach addressing both postural and respiratory dysfunctions in nonspecific low back pain management.

## Patient and public involvement

Patient and public involvement are not applicable.

This research did not receive any specific grant from funding agencies in the public, commercial, or non-profit sectors.

## Abbreviation:

NSLBP: non-specific Low back pain
FFT: finger to floor test
FVC: forced vital capacity
FEV1: forced expiratory volume in one second
PEF: peak expiratory flow
PPT: pressure pain threshold
ICC: intraclass correlation coefficient
BMI: body mass index
CA-Ax: amplitude coefficient axilar level
CA-Xif: amplitude coefficient xiphoid level
CA-ba: amplitude coefficient basal level
SPSS: statistical package for social sciences
SD: standard deviation
IBM: international business machines corporation
NY: New York
N: number of patients
COVID-19: Coronavirus SARS-CoV-2
